# True Kinematic Alignment Is Applicable in 44% of Patients Applying Restrictive Indication Criteria—A Retrospective Analysis of 111 TKA Using Robotic Assistance

**DOI:** 10.3390/jpm11070662

**Published:** 2021-07-15

**Authors:** Kim Huber, Bernhard Christen, Sarah Calliess, Tilman Calliess

**Affiliations:** 1 articon Spezialpraxis für Gelenkchirurgie, 3013 Berne, Switzerland; kimihuber@hotmail.com (K.H.); b.christen@articon.ch (B.C.); s.calliess@articon.ch (S.C.); 2 Campusradiologie Bern, Engeried-Spital, 3012 Berne, Switzerland

**Keywords:** individualized alignment, restricted kinematic alignment, robotic-assisted TKA, MAKO, safe zone, total knee arthroplasty

## Abstract

Introduction: Image-based robotic assistance appears to be a promising tool for individualizing alignment in total knee arthroplasty (TKA). The patient-specific model of the knee enables a preoperative 3D planning of component position. Adjustments to the individual soft-tissue situation can be done intraoperatively. Based on this, we have established a standardized workflow to implement the idea of kinematic alignment (KA) for robotic-assisted TKA. In addition, we have defined limits for its use. If these limits are reached, we switch to a restricted KA (rKA). The aim of the study was to evaluate (1) in what percentage of patients a true KA or an rKA is applicable, (2) whether there were differences regarding knee phenotypes, and (3) what the differences of philosophies in terms of component position, joint stability, and early patient outcome were. Methods: The study included a retrospective analysis of 111 robotic-assisted primary TKAs. Based on preoperative long leg standing radiographs, the patients were categorized into a varus, valgus, or neutral subgroup. Initially, all patients were planned for KA TKA. When the defined safe zone had been exceeded, adjustments to an rKA were made. Intraoperatively, the alignment of the components and joint gaps were recorded by robotic software. Results and conclusion: With our indication for TKA and the defined boundaries, “only” 44% of the patients were suitable for a true KA with no adjustments or soft tissue releases. In the varus group, it was about 70%, whereas it was 0% in the valgus group and 25% in the neutral alignment group. Thus, significant differences with regard to knee morphotypes were evident. In the KA group, a more physiological knee balance reconstructing the trapezoidal flexion gap (+2 mm on average laterally) was seen as well as a closer reconstruction of the surface anatomy and joint line in all dimensions compared to rKA. This resulted in a higher improvement in the collected outcome scores in favor of KA in the very early postoperative phase.

## 1. Introduction

In the effort to improve total knee arthroplasty (TKA), there is a growing interest in customizing alignment to the patient’s individual anatomy and soft-tissue balance. This is discussed as an alternative to the established standard mechanical alignment (MA) that has the same target for everybody. However, this individualized alignment is often not clearly defined, and a multitude of different philosophies, approaches, and terms can be found in the literature [1]. The clearest defined concept with the best clinical evidence available is the kinematic alignment (KA) approach [2]. The defined primary aim of KA is to reconstruct the individual joint surface of the femoral condyles with the prosthesis and thus to co-align the motion axes of the device to that of the patient’s knee [3]. Several studies report equal or even superior clinical outcomes compared to standard MA [4]. However, there are still critical voices. One point of criticism is about the resulting overall limb alignment and joint line obliquity in KA TKA. As the pre-arthritic situation is reconstructed, the components may be aligned in a significant varus or valgus angulation [5]. These deviations to the mechanical axis might lead to early implant failure. Further. it is questioned whether it makes sense to reconstruct pathologic situations that have led to osteoarthritis, or to better correct these with surgery. Another point of criticism is whether it is possible to properly reconstruct the individual joint surface with the use of a standard, symmetric prosthesis or if the different patient morphologies might require an individualized prosthesis [6,7]. Thus, not all patients may be suitable for KA TKA.

These concerns have led to an adaption of KA with the possibility for intraoperatively made adjustments to stay within a defined target zone of postoperative limb alignment, or to correct pathologic situations—the restricted kinematic alignment (rKA) approach [1]. Currently, there is a lack of a clear definition of when (or how often) adjustments are needed, and for what patients or phenotypes. Furthermore, there are only very limited data on the consequences for the patient—in terms of alignment, stability, and outcome [8,9]. Additional to this, there now is the need for technological support of some sort to be able to (1) identify a situation that needs to be adapted, and (2) precisely transfer this plan to the patient [10].

In this context, image-based robotics appears to be a promising tool for individualizing alignment with the concept of KA and rKA in standard TKA. Preoperative imaging enables a three-dimensional (3D) preplanning of the alignment philosophy based on the patient’s knee morphotype. Intraoperative adaptions with respect to soft tissue stability are possible. The robotic assistance ensures a precise implementation of the planning. As a result, all relevant parameters are transparent and comprehensible and thus are available for further evaluation.

In our institution, we established a clear workflow for individualized alignment in robotic-assisted TKA since 09/2018. The starting point is the KA principle with certain self-defined alignment limits based on our experiences and the current literature [11]: (1) Resulting overall limb alignment of 176–181°, (2) a joint line obliquity up to 4° to the mechanical axis, and (3) a neutral rotation of the component trochlea groove to the anatomical trochlea axis. When these criteria could not be achieved with a KA-based component position, adjustments were made to a restricted KA, as described below.

Based on this, the research questions of this study were: (1) With said criteria, how often can we conduct a true KA in a standard TKA collective and how often do we have to make adaptions? (2) Are there differences in the suitability for KA (with proposed limits) between different knee morphotypes? (3) What are the differences between a true KA and an rKA in terms of alignment, resulting joint stability, and early postoperative outcome?

## 2. Methods

### 2.1. Patient Collective

The study includes a retrospective review of our institutional database on knee arthroplasties between 10/2019 and 04/2021. All patients were included who gave their informed consent to participate in the prospective data collection and received an image-based, robotic-assisted, primary unconstrained TKA for any indication in that time period. The patient demographics (age, sex, ASA score, and previous surgeries on the affected knee) and preoperative knee alignment were determined. On preoperative long leg standing X-rays (EOS System), the anatomical and the mechanical hip–knee–ankle angle (HKA) as well as the standard joint line angles (MPTA, mLDFA) were measured by an independent radiologist. Based on the determined anatomical femorotibial angle, three subgroups were defined: (1) Neutral (5–10°), (2) varus OA (<5°), and (3) valgus OA (>10°).

### 2.2. Application of Individualized Alignment Philosophies

All patients received an individualized alignment—true KA or rKA—based on their knee anatomy/morphotype, ligament situation, and the defined boundaries for KA following a standardized workflow.

**Figure 1:**
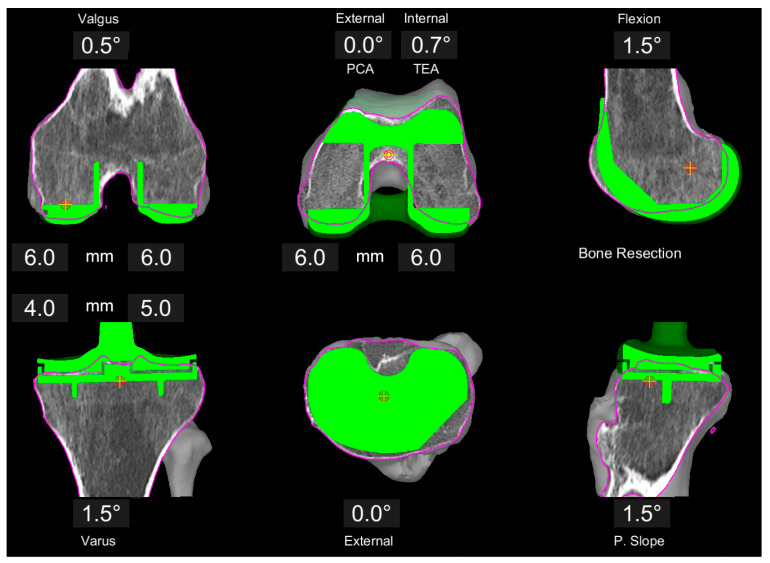
Example of image-based planning of the component position based on the principles of KA. Femoral resections are set to 6 mm each, resulting in 0.5° valgus position and 0.7° internal rotation with respect to the TEA. Tibia plan is preliminary at 1.5° varus, 1.5° slope and a resection level of 4 and 5 mm, respectively.

All surgeries were conducted with the MAKO robotic arm and the Triathlon PS knee system (Stryker, Kalamazoo, MI, USA). Prior to surgery, three-dimensional preplanning of the individualized component position using the proprietary MAKO software was conducted by the operating senior surgeon following the concept of kinematic alignment (KA) (Figure 1). First, the distal and posterior resections on the femur were set symmetrically at 6 mm bone resection. This results in an individual distal femoral angulation and a 0° rotation with reference to the posterior condylar axis (PCA) (individual rotation to transepicondylar axis (TEA)). In a second step, the femoral component size was defined to best reconstruct the anterio-posterior and medio-lateral dimensions without producing an overhang. Based on this position and size, the femoral flexion was adjusted to create a smooth anterior transition without notching.

The tibia orientation was only preliminarily planned, starting with a rather conservative orientation for varus/valgus, slope, and resection level, and finally determined during surgery based on the soft tissue situation in order to achieve a symmetrically balanced extension space and an isometric gap in the medial compartment (see below).

**Figure 2:**
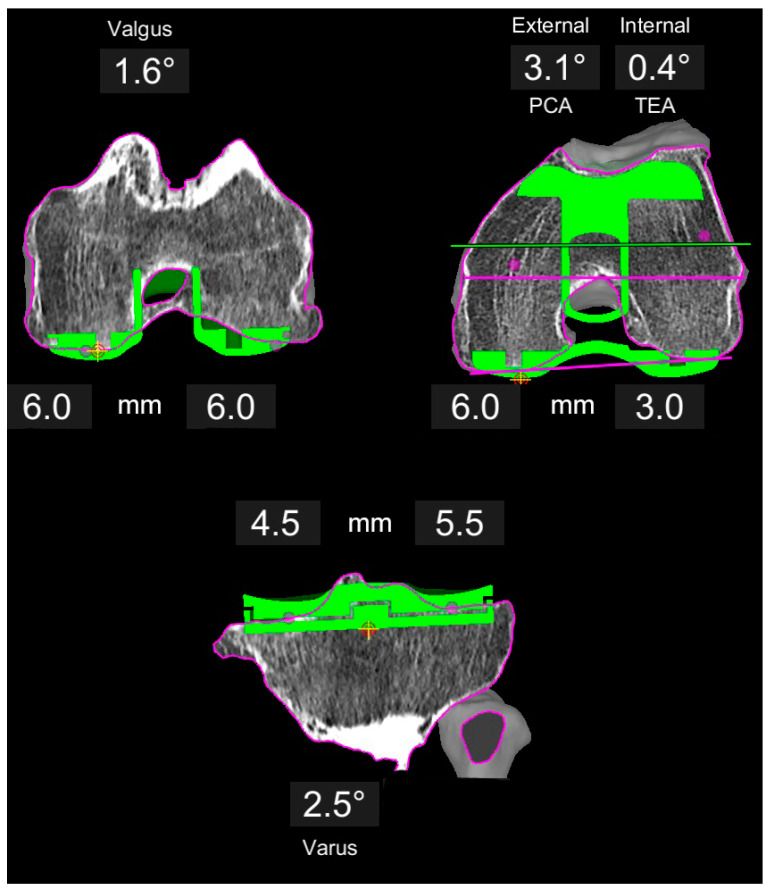
Example of a varus knee planned with restricted KA. The distal femoral resection is symmetric at 6 mm, whereas the posterior resection is adjusted to co-align the trochlea groove close to the native trochlea axis. This results in a 3.1° external rotation with respect to the PCA. Tibia follows the same principle as for KA (Figure 1).

**Figure 3:**
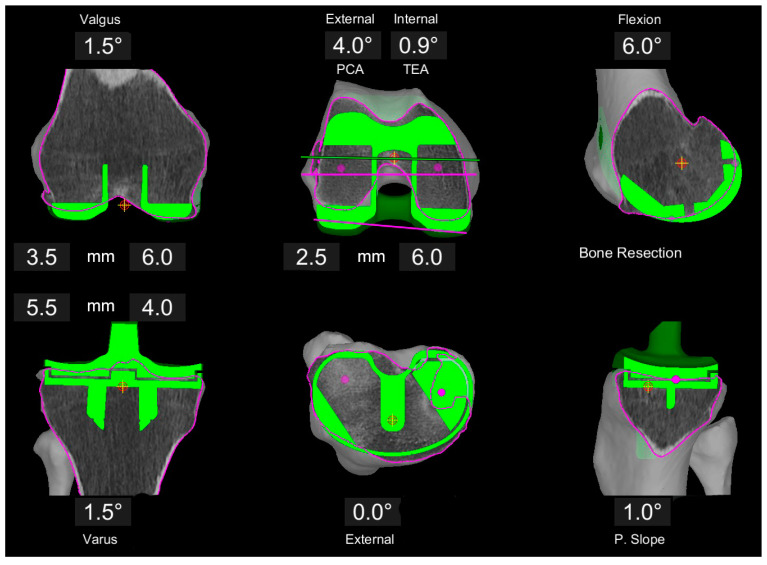
Example of image-based planning of the component position based on the principles of rKA in a valgus patient. Medial femoral resections are set to 6 mm each, whereas the lateral resections are adjusted. With the tibia in 1.5° varus, a 1.5° valgus on the femur is set to create a neutral overall limb alignment in the coronal plane. Femoral rotation is set to best reconstruct the trochlea axis with the component trochlea groove resulting in 4° external rotation to the PCA (0.9° internal to TEA).

Adaptions to this KA planning were made if the symmetric posterior resection on the femur resulted in relative malrotation of the component trochlea groove (usually internal) with respect to the native trochlea axis. In these cases, the femoral rotation was adjusted with more external rotation around a medial pivot point (constant medial posterior resection at 6 mm, less lateral post resection) (Figure 2). Adaptions to the distal femoral resection/orientation were made if the distal femoral angle deviated more than 4° from the mechanical axis or the resulting overall limb alignment was greater than 1° valgus. Again, the medial femoral resections remained at 6 mm (concept of anatomic reconstruction of the medial column), whereas the lateral resection was reduced (Figure 3). The limit for the tibia component varus and the overall varus limb alignment was 4° deviation to the mechanical axis. All these adaptions were classified as a restricted kinematic alignment (rKA) if the difference between medial and lateral resection on the femur was greater than 1 mm, or if a soft tissue release other than resection of the osteophytes and posterior capsule release was necessary to achieve balanced gaps. All other cases were defined as true KA.

The pre-planned resection level was verified intraoperatively based on the individual cartilage thickness of the knee (when available) and adjusted accordingly. Therefore, the cartilage level was added to the CT-model of the knee using the blunt probe. After that, a robotic-assisted precut of the tibia (based on the conservative preplan) was made to access and resect all relevant osteophytes affecting the soft tissue envelope. In cases of relevant extension deficit or massive posterior osteophytes, the distal and posterior femoral osteotomies were also conducted to ensure complete osteophyte removal and posterior capsule release. Additionally, both cruciate ligaments were resected prior to soft tissue analysis.

Then, a spreader was introduced in the knee to reproducibly record the extension and flexion space. Based on the predicted gap width and symmetry, the definite tibia alignment was determined to create a stable extension space at 18–19 mm medial and lateral, and the same width for the medial flexion gap ±1 mm. Mainly the varus/valgus orientation and the resection height were adjusted, and seldom the tibia slope if a flexion/extension mismatch was present. The lateral flexion gap was recorded but left as it was, unless it had been tighter than the medial. The distal femoral alignment was not adjusted, and only the femoral rotation in said rare cases. Soft tissue releases were used only if the mentioned boundaries for the overall limb alignment were reached.

This final implant position was then transposed to the knee with the help of the robotic arm. After insertion of the definite prosthesis, the final alignment (varus/valgus orientation of femur and tibia, femoral rotation, bone resection levels) and the joint stability (medial and lateral spaces) in 0° extension and 90° flexion were recorded using the robotic software.

### 2.3. Comparative Analysis between KA and rKA

Intraoperative robotic data on the resulting prosthesis alignment, resection levels, joint spaces, or necessary soft tissue releases were recorded as described above. The parameters of interest were analyzed in a descriptive statistical analysis using Microsoft Excel for Mac Version 15.34. The mean values as well as the range and standard deviation were calculated using the standard excel formulas. Additionally, a standardized outcome measurement using the Knee Injury and Osteoarthritis Outcome Score (KOOS), the Knee Society Score (KSS), the Oxford Knee Score (OKS), and EQ-5D was carried out preoperatively and at 2-months follow-up. Because of different preoperative mean values between the subgroups, only the improvement from preoperative to postoperative (delta between the scores) was analyzed in this study.

## 3. Results

### 3.1. Patient Collective

During the defined time period, 317 primary knee arthroplasties were performed in our institution. One hundred and forty (45%) were partial knee prosthesis (UNI), 12 (4%) were primary hinge-type prostheses, and 36 (11%) were conventional primary unconstraint TKA. The remaining 126 TKAs were performed with robotic arm assistance, out of which 111 could be included in the study (patient’s consent). Patients’ demographics are displayed in Table 1. The preoperative alignment parameters and distribution of the subgroups are displayed in Table 2.

### 3.2. Application of Individualized Alignment Philosophies

In total, 49 patients (44%) received a true KA, whereas in 62 cases (56%), adjustments to an rKA were made. As displayed in Table 3, the suitability for KA meeting our selection criteria differed between the subgroups with 70% in varus patients, 25% in the neutral alignment group, and 0% in the valgus group.

### 3.3. Comparative Analysis between KA and rKA

In the KA group, no soft tissue releases were necessary to achieve balanced gaps. In the rKA group, releases were made in 11 cases (18%). In the rKA varus subgroup, major adjustments were made for the femoral rotation to co-align the prosthesis to the trochlea axis. In 15 of the 21 varus rKA cases, an asymmetric posterior resection was conducted. Adaptions in the coronal plane were only made in seven of the varus cases to stay within the limits of ±4° deviation to the mechanical axis. Of the 21 rKA varus cases, 2 needed additional soft tissue release (resection of medial tibia plateau and downsizing tibia, 10%).

In contrast to this, the rKA adjustments in the valgus subgroup often affected both distal and posterior asymmetric resection levels (17 of 23 cases). Seven of the valgus cases needed a soft tissue release laterally (30%).

The intraoperative alignment parameters of the components and the resulting joint spaces are displayed in Table 4. The greatest variability in the alignment was in terms of component rotation ranging from 4° internal to 3.2° external rotation with respect to the TEA. There was no difference between KA and rKA alignment parameters in the varus or neutral subgroups, except for the resection levels on the lateral condyle so they are displayed together.

At 2-months follow-up, the KA group showed greater improvement and faster rehabilitation in every measured outcome parameter compared to the rKA group, as displayed in Table 5.

## 4. Discussion

The first question was how often is KA applicable in TKA and how often are adaptations to rKA necessary? The most important finding was that in our TKA collective, “only” 44% of the patients were suitable to receive a true KA, whereas in 56%, adjustments to a symmetric resection on the distal and posterior femur were necessary to stay within our defined boundaries of overall limb alignment (HKA 176–181°) and joint line orientation ±4° or to reconstruct the anatomic trochlea groove. In the current literature, there are little comparable data to this. Almaawi et al. conducted an analysis of CT data and defined the boundaries of KA ±5° for the component position in the coronal plane and ±3° for the overall limb axis [9]. In their collective, 51% of the patients were suitable for KA without adaptions. However, it is unclear what impact possible bone defects had, that might influence measurement especially on the tibia. Only bone anatomy in the coronal plane and not the soft tissues or the axial plane were included in the analysis. Other studies aiming for true KA (with or without limits) report on necessary soft tissue releases in 7–33% of the cases [12,13,14]. However, it remains unclear in how many cases adjustments to KA were made. With “only” 10% soft tissue releases in the whole collective (18% in the rKA group, none in the KA group), we are at the lower end of the literature.

Nevertheless, different aspects have to be considered when evaluating the numbers presented in our study. First, our indication limits for true KA were more restrictive, especially for valgus patients compared to the mentioned literature. We did not accept more than 1° valgus deviation for the postoperative overall limb axis. This is because many valgus patients have additional pathologies at the hip and ankle joint increasing the functional valgus during gait. This has a higher risk for implant failure and secondary medial instability. In addition, the benefit of KA for valgus patients has less evidence compared to varus morphotypes. Taking a closer look at outcome data for KA versus MA, studies without indication limits for valgus patients tend to have a minor positive effect compared to those with restrictive indications [15,16]. This explains 100% of rKA in the valgus group and 75% in the neutral group—by our definition, the mild constitutional valgus patients (average LDFA 87°, MPTA 89° = 2° constitutional valgus on average). Second, our institutional proportion of uni- and bi-uni-compartmental knee arthroplasties is at 45%. Thus, our current collective for TKA probably overrepresents severe deformities, posttraumatic situations, and secondary OA after correction osteotomies. Fifty-five percent of our patients had previous surgeries on their affected knee. Lastly, all currently available studies setting limits for KA concentrate on the coronal limb alignment only as inclusion/exclusion criteria [12,17,18]. In contrast to this, we also included the axial plane and the reconstruction of the physiological trochlea axis with the prosthesis. Especially in the varus group, most of the adjustments that were conducted were made to address the rotational orientation of the femur to meet the trochlear anatomy. Riviere et al. had already raised the question of this being a critical point in KA when using standard symmetrical implants [19]. In clinical research, an equal patellofemoral complication rate is found with true KA and MA [20]. Furthermore, biomechanical research reports on a high variability on the patellofemoral kinematics in KA TKA [21]. Thus, our intension was to address this issue by optimizing the three-dimensional orientation of the component. This had a significant effect on the percentage of rKA in the varus group. On the other hand, only in seven varus patients was a correction in the coronal plane necessary.

Regarding our research question (3), we were able to display slight differences in the resulting final component alignment between the subgroups. The varus group showed an LDFA between 87° and 91° with 89.2° on average and an 87.8° MPTA (range 86°–89°). This is close to what would be expected for varus patients [22]. The valgus group differed more than what we expected. This is the result of the rKA, aiming for a neutral overall limb axis. In these classic valgus patients, the tibia joint line is much more horizontal and thus adaptions of the LDFA were necessary for the desired correction. As a result, the physiological morphotype is altered most in this valgus collective.

The overall greatest variability was seen for the component rotation (both in true KA and rKA groups) with on average 0.7° internal rotation with respect to the TEA. This completely differs from the mechanical alignment philosophy with systematic external rotation of the component by 3° in the measured resection principle. However, our findings are consistent with the current literature analyzing the native knee anatomy. Vercruysse et al. described a broad variability between the anterior trochlea line and the TEA, with internal rotation on average [23].

The last point to emphasize is the resulting joint stability created with different alignment philosophies. Whereas rKA in valgus patients on average resulted in equal and symmetric flexion and extension spaces, varus KA patients showed a symmetric extension gap, but a more trapezoidal flexion gap. The average difference between the medial and lateral flexion compartment was 2 mm and ranged 0–5 mm. Moreover, the medial flexion gap had a higher standard deviation, tending toward more laxity, compared to valgus patients. The resulting spaces are thus more physiologically reproduced compared to the native knee situation [24]. In addition, this finding is consistent with other literature analyzing the gaps in KA TKA [14,25]. Peter McEwen et al. described differences in the medial and lateral flexion gap up to 8 mm.

In the patients’ outcome measurements, we found a higher improvement in all outcome scores from preoperative to 2 months follow-up in favor of the KA group. This is consistent with the literature [16]. Possibly, this is a result of the more anatomic position of the implant and more physiological soft tissue balance already improving the initial rehabilitation. However, the outcome data should not be over-interpreted as only a very short follow-up period is included and there were some differences in the preoperative scores between the subgroups.

The study has several limitations. First, it is a retrospective analysis of a preselected collective. Patients assigned for uni-compartmental prosthesis (45% in our institution), or primary hinge-type prosthesis (4%) were not included in the study. Second, the criteria to choose between KA and rKA is artificial and might differ to other institutions. Moreover, the trochlea axis, which was used to adjust the femoral component rotation, is not super precisely defined. Thus, minor deviations are possible. Third, no long-term outcome data are included in the analysis. Accordingly, no definite statement can be made as to whether the decision in favor of KA or rKA made sense and was beneficial for the patient.

## 5. Summary and Conclusions

In this study, we reviewed alignment parameters in TKA patients treated with an individualized alignment following the principles of KA or rKA. With a progressive indication for UNIs over TKA, and boundaries for KA in terms of overall limb axis, joint line obliquity, and rotation, only 44% of the cases were applicable for a true KA. In the KA group, a more physiological knee balance reconstructing the trapezoidal flexion gap was seen as well as a closer reconstruction of the surface anatomy and joint line in all dimensions. In the varus group, interestingly, the limiting factor for KA was less than the coronal plane and overall varus angulation than the axial plane with reconstructing the trochlea groove physiologically. Although, 70% of the varus patients were planned for KA TKA. In the valgus group, adjustments were usually made in both the coronal and axial plane and 100% were adjusted to an rKA. The initial rehabilitation phase showed a greater improvement in the KA group compared to the rKA group in all analyzed outcome scores.

Now, the interesting focus for the future is to compare the effect of adjustments on the patient´s long-term outcome; however, our data suggest that we are talking about different starting points and phenotypes that might not be perfectly comparable.

## Figures and Tables

**Table 1:** Patients´ demographics of total collective.

age (range)	68 years (40–87)
sex	42 male/69 female
ASA score (I/II/III)	6%/73%/21%
previous surgeries:	
no	50 (45%)
meniscectomy	33 (30%)
ligament reconstruction	17 (15%)
patella re-alignment	15 (14%)
tibia osteotomy	8 (7%)
fracture osteosynthesis	4 (4%)
other	3 (3%)

**Table 2:** Preoperative alignment parameters of total collective and subgroups.

	Total (*n* = 111)	Neutral Group (*n* = 28)	Varus Group (*n* = 60)	Valgus Group (*n* = 23)
Mean HKA (range)	177° (161–196)	180° (175–184)	171° (161–178)	188° (182–196)
Mean MPTA (range)	87° (80–93)	89° (85–92)	86° (80–89)	89° (86–93)
Mean mLDFA (range)	88° (81–92)	87° (84–90)	89° (84–92)	85° (81–88)

HKA = hip-knee-ankle angle, MPTA = medial proximal tibia ankle, mLDFA = mechanical lateral distal femur ankle.

**Table 3:** Distribution of alignment philosophy chosen for each subgroup.

	Neutral Group (*n* = 28)	Varus Group (*n* = 60)	Valgus Group (*n* = 23)
True KA	25% (*n* = 7)	70% (*n* = 42)	0%
Restricted KA	75% (*n* = 21)	30% (*n* = 18)	100% (*n* = 23)

**Table 4:** Final set component alignment intraoperatively and final gap symmetry.

	Neutral Group (*n* = 28)	Varus Group (*n* = 60)	Valgus Group (*n* = 23)
Mean tibia comp. varus (range)	1.8° (0–4°)	2.2° (1–4°)	1.2° (0–2.5°)
Mean femur comp. valgus (range)	1.4° (0–4°)	0.8° (−1–3°)	1.1° (0–2.5°)
Mean rotation to TEA (range)	−0.7° (=internal) (−3–1.5°)	−0.7° (−3.9–3.2°)	0.1° (−3–3°)
Mean diff. med. to lat. extension space (std. dev.)	0 mm (±0.46 mm)	0 mm (±0.57 mm)	0 mm (±0.52 mm)
Mean diff. med ext. to flex. space (std. dev.)	0 mm (±1.03 mm)	0 mm (±1.32 mm)	0 mm (±0.98 mm)
Mean diff. med. to lat. flexion space (std. dev.)	1 mm (±1.26 mm)	2 mm (±1.79 mm)	0 mm (±1.38 mm)

Comp. = Component, diff. = difference, med. = medial, lat. = lateral, ext. = extension, flex. = flexion, std.dev. = standard deviation.

**Table 5:** Improvement in outcome measurement from pre-operative to 2-months follow-up (delta) for KA and rKA group.

Score	KA Group (*n* = 49)	rKA Group (*n* = 62)
KOOS		
Symptoms	+26 pts	+17 pts
Pain	+23 pts	+20 pts
ADL	+24 pts	+20 pts
Sports	+22 pts	+16 pts
QOL	+37 pts	+30 pts
Knee Society Score	+61 pts	+55 pts
Oxford Knee Score	+10 pts	+7 pts
EQ-5D	0.25	0.13
